# Recent novel approaches to limit oxidative stress and inflammation in diabetic complications

**DOI:** 10.1002/cti2.1016

**Published:** 2018-04-18

**Authors:** Raelene J Pickering, Carlos J Rosado, Arpeeta Sharma, Shareefa Buksh, Mitchel Tate, Judy B de Haan

**Affiliations:** ^1^ Department of Diabetes Central Clinical School Monash University Melbourne VIC Australia; ^2^ Oxidative Stress Laboratory Basic Science Domain Baker Heart and Diabetes Institute Melbourne VIC Australia; ^3^ Heart Failure Pharmacology Basic Science Domain Baker Heart and Diabetes Institute Melbourne VIC Australia

**Keywords:** cardiovascular disease, chronic kidney disease, diabetes, inflammation, oxidative stress, retinopathy

## Abstract

Diabetes is considered a major burden on the healthcare system of Western and non‐Western societies with the disease reaching epidemic proportions globally. Diabetic patients are highly susceptible to developing micro‐ and macrovascular complications, which contribute significantly to morbidity and mortality rates. Over the past decade, a plethora of research has demonstrated that oxidative stress and inflammation are intricately linked and significant drivers of these diabetic complications. Thus, the focus now has been towards specific mechanism‐based strategies that can target both oxidative stress and inflammatory pathways to improve the outcome of disease burden. This review will focus on the mechanisms that drive these diabetic complications and the feasibility of emerging new therapies to combat oxidative stress and inflammation in the diabetic milieu.

## Introduction

The global prevalence of diabetes mellitus (DM) is estimated to be 415 million people (8.8% of the world population) with a total health expenditure of 673 billion dollars.^1^ The number of people with DM is estimated to increase to 642 million people in the next 25 years, with an expected expenditure of 802 billion dollars. This expanding global epidemic is mostly being driven by an increase in the number of patients presenting with type 2 diabetes (T2D), a disease fuelled by risk factors such as western diet‐linked obesity, metabolic syndrome, sedentary lifestyle, and high blood pressure. On the contrary, type 1 diabetes (T1D) occurs as a result of autoimmune destruction of the insulin‐producing cells of the pancreas. Despite the origin and timing of disease onset differing between patients with T1D and T2D, both groups exhibit elevations in blood glucose, leading to end‐organ injury. Elevations in risk factors such as blood glucose and blood pressure are mostly well controlled, with DM patients often requiring several medications to lessen these risks. However, despite the increased understanding and management of T2D, complications associated with DM continue unabated, leading to significant morbidity and mortality. In particular, diabetic patients are at a much higher risk of developing micro‐ and macrovascular complications than the nondiabetic population. Therefore, a better understanding of diabetes‐associated complications and development of improved and targeted therapies is warranted. This review will highlight four significant vascular complications associated with DM. It will examine the molecular origins leading to these complications, with a focus on oxidative stress and inflammation, two interconnected mechanisms now being recognised as significant drivers underpinning these complications. Novel therapeutic approaches are emerging, based on these newer avenues of exploration. These will be discussed and placed in the context of feasible drug strategies going forward.

## Reactive oxygen species and oxidative stress

Reactive oxygen species (ROS) have been proposed as significant drivers of diabetic complications. The central mechanism mediating these diabetic complications initially focused on dysregulated ROS production by the mitochondria.^2^ Mitochondria are involved in essential biological processes including the production of ATP by oxidative phosphorylation (OXPHOS), calcium homeostasis, and regulation of cell death. OXPHOS involves the transfer of electrons across complexes I–IV of the electron transport chain resulting in the generation of membrane potential (Δψ_m_) that drives the proton motive force of ATP Synthase. A by‐product of OXPHOS is the generation of the ROS superoxide (O_2_^**.**−^) that occurs as electrons escape complexes I and III and react with molecular oxygen.^3^ Although ROS are a by‐product of the normal metabolism of oxygen produced by mitochondria, diabetes results in oxidative stress as a consequence of altered mitochondrial ROS production and clearance.^4^

In addition, particular enzymes such as xanthine oxidase, lipoxygenase, myeloperoxidase, and NADPH oxidase (NOX) also produce ROS. There is evidence for increased enzymatic activity and ROS‐mediated damage in diabetes. For example, the activity of xanthine oxidase is increased in experimental T1D^5^ and more recently shown to be elevated in diabetic patients with peripheral neuropathy.^6^ High glucose is able to enhance activation of the 12/15‐lipoxygenase pathway in vascular smooth muscle cells and endothelial cells.^7^, ^8^ P22phox and the NOX isoforms, NOX1 and NOX2, form a complex and use NADPH as the substrate to transfer electrons from NADPH to oxygen to generate O_2_^**.**−^. The NOX4 isoform is considered as the hydrogen peroxide generating isoform. One mechanism leading to enhanced NOX activity includes hyperglycaemia‐driven formation of intermediate Amadori products which are oxidised to form AGEs that activate RAGE and stimulate NOX to produce ROS.^2^ A clear role for elevated NOX1 activity has been shown in the promotion of diabetes‐associated atherosclerosis,^9^ whilst NOX4 appears critical in driving diabetic nephropathy.^10^

Reactive oxygen species are additionally produced when endothelial nitric oxide synthase (eNOS) becomes uncoupled and instead of generating NO, eNOS produces superoxide anions. Under physiological conditions, insulin stimulates eNOS‐induced production of nitric oxide (NO) via the PI3‐kinase/AKT pathway. Several mechanisms have been proposed to account for the uncoupling of eNOS. Protein kinase C (PKC) and AGEs, known to be activated and increased by diabetes, respectively, induce eNOS uncoupling via post‐translational modifications in the vascular wall. In particular, diabetes‐driven inactivation of phosphorylated eNOS (on Ser‐1177), mediated via *O*‐linked attachment of β‐*N*‐acetylglucosamine (*O*‐GlcNAc), has been shown to cause eNOS uncoupling and reduced NO formation in endothelial cells.^11^ Additionally, the oxidation of tetrahydrobiopterin (BH4), an essential cofactor utilised by eNOS to form NO, leads to eNOS uncoupling. Specifically, BH4 oxidation by the reactive oxygen species, peroxynitrite (produced through the interaction of O_2_^**.**−^ with NO), leads to irreversible eNOS enzyme inhibition.^12^ Another mechanism includes the glucose‐mediated oxidation of the zinc‐tetrathiolate cluster in eNOS leading to increased O_2_^**.**−^ formation.^13^

The cellular concentration of ROS is determined by the rate of production verses rate of clearance. ROS are targeted for removal by antioxidants and enzymes such as superoxide dismutases (SOD), catalase, glutathione peroxidases (GPx), peroxiredoxins, and thioredoxins. In preclinical diabetic rodent models and in diabetic patients, the expression of antioxidants such as GPx has been shown to be reduced resulting in increased markers of oxidative damage.^14^, ^15^, ^16^

Despite the origins of diabetes‐driven ROS production and accumulation, it has been proposed that elevations in ROS inhibit glyceraldehyde 3‐phosphate dehydrogenase (GAPDH).^2^ This in turn affects major downstream metabolic pathways including PKC, the receptor for advanced glycation end‐products (RAGE)/AGE axis, the renin–angiotensin–aldosterone system (RAAS), and the hexokinase and polyol pathways.^2^ Dysregulation of these major metabolic pathways has been linked to diabetes‐associated micro‐ and macrovascular complications.^2^ In summary, hyperglycaemia leads to elevated ROS, which dysregulates important metabolic pathways to promote diabetic vascular disease.

## Diabetes and cardiovascular disease

Atherosclerosis is a focal disease caused by the deposition of oxidised low‐density lipoprotein (ox‐LDL) cholesterol within the vessel wall. This leads to the accumulation of plaque in the coronary arteries that supply blood to the heart or within the aortic arch carrying blood away from the heart. In particular, activated macrophages scavenge ox‐LDL to form foam cells^17^ that together with cholesterol crystals form stable and unstable plaque, leading to vessel obstruction and plaque rupture, respectively.^18^, ^19^ This process results in the narrowing of the arteries specifically at arterial branch points or bifurcations subjected to altered blood flow. Diabetic patients are 2–4 times more likely to develop major vascular complications as a consequence of atherosclerotic plaque build‐up within their arteries.^20^ Diabetic patients are additionally more prone to plaque rupture.^21^ It is now well established that the morphology of the diabetic plaque differs with respect to its cellularity when compared with nondiabetic patients. Most noticeable is the greater macrophage and T lymphocyte infiltration into the atherosclerotic plaque, as well as larger necrotic cores compared with nondiabetic specimens.^22^ With increased severity comes an associated increased risk of coronary artery disease, myocardial infarction, stroke, or peripheral artery disease.^23^, ^24^ Atherosclerotic cardiovascular disease (CVD) remains the principal cause of death and disability in people with DM, especially in people with T2D.^25^ It is estimated that CVD‐associated events occur 14.6 years earlier in people with DM than in people who do not have DM.^26^

### Endothelial dysfunction

**Figure 1:**
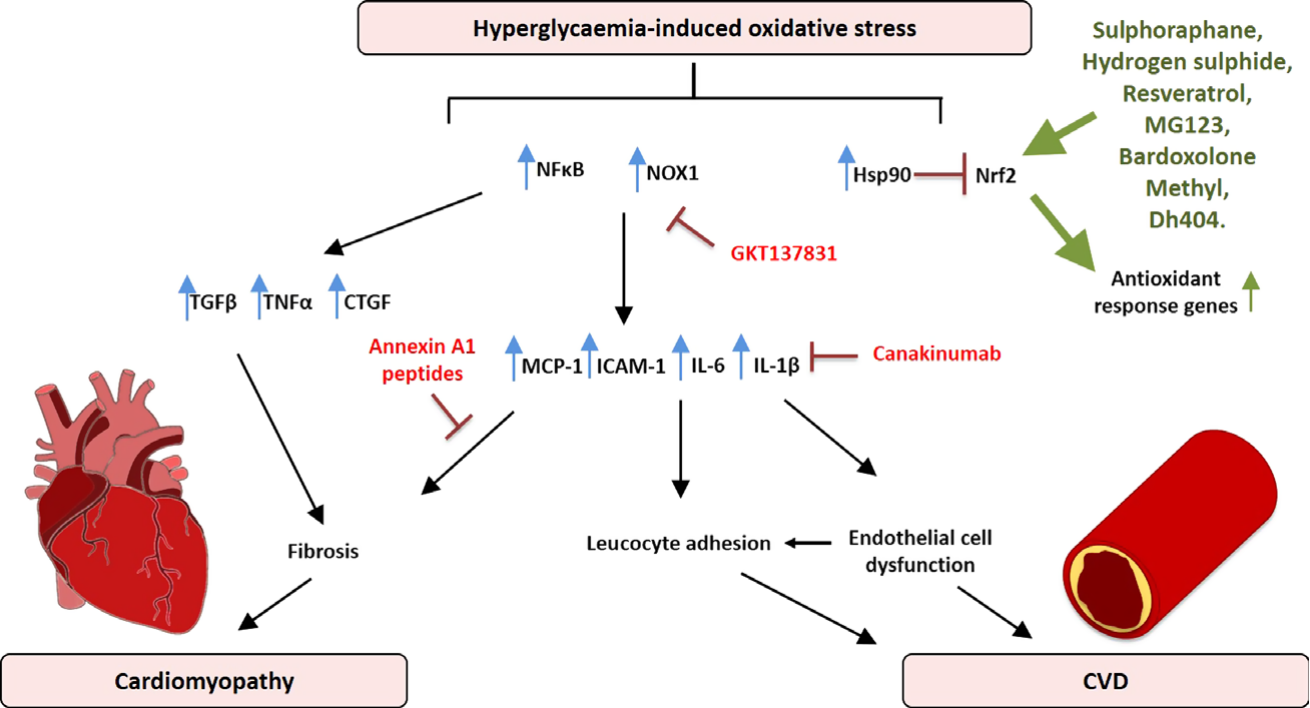
A schematic diagram demonstrating the mediators involved in hyperglycaemia‐induced oxidative stress leading to the progression of diabetic cardiomyopathy and cardiovascular disease (CVD). Oxidative stress leads to the upregulation of nuclear factor kappa B (NFκB), NADPH oxidase (NOX1), heat‐shock protein 90 (Hsp90), transforming growth factor beta (TGFβ), tumor necrosis factor alpha (TNF‐α), connective tissue growth factor (CTGF), monocyte chemotactic protein 1 (MCP‐1), intercellular adhesion molecule 1 (ICAM‐1), interleukin 6 (IL‐6) and interleukin 1β (IL‐1β). In red is the NOX oxidative enzyme inhibitor GKT137831, the Il‐1β inhibitor (Canakinumab) and annexin A1 peptides, whereas in green are the Nrf2 activators (sulphoraphane, hydrogen sulphide, resveratrol, MG123, bardoxolone methyl and dh404) that have shown protective effects in diabetic cardiac and vascular disease.

A common hallmark of both T1D and T2D leading to atherosclerosis is the attenuation of endothelial function.^27^, ^28^ A dysfunctional endothelium is considered an independent risk factor for CVD which promotes leucocyte and platelet adhesion, thrombosis, and inflammation, all of which are critical steps in the progression of atherosclerosis.^29^ Altered mitochondrial dynamics, in particular mitochondrial fission, can result in a net increase in the production of ROS.^30^ This has been observed in venous endothelial cells from patients with DM, and in cultured cells exposed to 30 mm glucose, and supports the observation that mitochondrial ROS generation is increased by diabetes.^30^ In addition, uncoupled eNOS and the activation of NOX isoforms are associated with diabetes‐associated endothelial dysfunction.^31^ ROS such as superoxide, hydrogen peroxide, and peroxynitrite have been shown to be produced by various cells of the vasculature including endothelial and vascular smooth muscle cells.^32^ Indeed, oxidative stress is now considered a significant driver of endothelial dysfunction (Figure 1).

### The NADPH oxidase proteins

NADPH oxidases are expressed in monocytes, macrophages, and cells of the vasculature.^33^ NOX1 and NOX4 are expressed in the walls of the vasculature, whilst NOX2 and NOX4 are present in endothelial cells.^34^ In separate studies using a mouse model, genetic deletion of NOX1 led to a decrease in atherosclerosis development using either an atherogenic diet (to induce T2D) or streptozotocin (STZ) (to induce T1D).^9^ Deletion of NOX2 has no effect on lesion development in the aortic sinus, but reduced lesion size in the descending aorta.^35^ Deficiency in p47phox, which regulates both NOX1 and NOX2, leads to a decrease in atherosclerosis.^36^ Interestingly, NOX4 appears to be protective in the aorta as deletion or inhibition of NOX4 resulted in increased atherosclerosis associated with increased inflammation in both vascular and immune cells.^37^ Activation of PKC and the generation of AGEs, both of which are upregulated in the diabetic environment, have been shown to activate NOX isoforms in monocytes and macrophages, and drive an increase in the expression of pro‐inflammatory genes such as IL‐6, monocyte chemoattractant protein 1(MCP‐1), and intercellular adhesion molecule 1 (ICAM‐1).^38^, ^39^ By activation of these pathways, it has been demonstrated that inflammation is increased throughout the body, leading to the generation of ROS.

### Endothelial nitric oxide synthase and nitric oxide

Endothelial NO protects against atherosclerosis by regulating cellular proliferation, leucocyte adhesion, and platelet aggregation. Uncoupled eNOS leads to decreased NO production by endothelial cells, but also potentiates oxidative stress in the vasculature, which promotes atherosclerosis.^40^ In diabetic settings, the uncoupling of eNOS and resultant superoxide production is well documented leading to impaired vascular repair and atherosclerosis, an effect that is rescued with the administration of NO stimulating peptides.^41^

### Antioxidants as targets in clinical trials

A large body of literature based on *in vitro* experiments and *in vivo* animal trials provides evidence that antioxidants play a key role in the reduction of ROS. This suggests that antioxidants would be valuable therapeutics to reduce the homeostatic dysregulation caused by chronic oxidative stress and promoted by DM. Clinical studies have used antioxidant therapeutics based on the preclinical evidence of the harmful effect of prolonged oxidative stress and inflammation in the development of atherosclerosis. These studies include the Women's Health Study, The Women's Antioxidant Cardiovascular Study and The Physicians’ Health Study II.^42^, ^43^, ^44^ In all cases, antioxidant vitamin supplementation, which had been shown to work in preclinical studies, was insufficient to produce a significant net‐positive effect in the treatment or management of CVD. This discrepancy between the preclinical and clinical trial data has been explained by the dose administered, the relatively poor uptake of vitamins by target organs, interference of other medications, genetic and environmental factors, and severity of disease. Vitamin E supplementation remains inconclusive with one meta‐analysis of 49 studies demonstrating a significant reduction in cardiovascular mortality risk using supplements containing Vitamin E,^45^ whilst another study showed that the use of high‐dose vitamins, including vitamin E, led to an increase in all‐cause mortality,^46^ suggesting that fine‐tuning of the vitamin dose is important, especially given the knowledge that ROS are essential for metabolic signalling. Newer research is now focused on the development of small‐molecule inhibitors or antibodies, with the capacity to target specific receptors involved in signal pathways involved in the generation of ROS, to upregulate the antioxidant response under conditions of oxidative stress.^47^, ^48^

### Targeting inflammation to limit atherosclerosis in the CANTOS trial

The CANTOS Trial used canakinumab, a therapeutic monoclonal antibody targeting interleukin‐1β (IL‐1β). 10,061 patients were enrolled into the trial with previous myocardial infarction and a high‐sensitivity C‐reactive protein (Hs‐CRP) level above 2 mg L^−1^.^49^ Subjects were divided into four groups, three of which received doses of canakinumab (50, 150 or 300 mg), while the final group received a placebo. The CANTOS trial determined that the targeting of the IL‐1β signal pathway with a dosage of 150 mg canakinumab led to a significantly lower rate of recurrent cardiovascular events compared to placebo, and this was independent of an effect on lipid‐lowering.^49^

### Targets for future research

#### Nrf2 – The master regulator of antioxidant responses

Nuclear factor erythroid‐2 (E2)‐related factor 2 (Nrf2) is a transcription factor that acts as the master regulator of oxidative stress. Nrf2 is expressed in vascular endothelial cells, and controls basal and induced levels of antioxidants such as heme‐oxygenase‐1 (HO‐1), NAD(P)H dehydrogenase quinone‐1 (NQO‐1), SOD, GPx and detoxifying enzymes. Mechanistically, Nrf2 is held in an inactive complex in the cytosol through interaction with its negative regulator, Kelch‐like ECH‐associated protein 1 (Keap1), where it undergoes ubiquitination and subsequent turnover by the proteasome through interaction with Keap1 and Cullin3. Alternatively, in response to oxidative stress, Keap1 undergoes oxidative modification and Nrf2 is released from Keap1.^50^ Nrf2 then translocates into the nucleus where together with a small‐molecule regulator Maf1, it binds to antioxidant response elements (ARE) in the promoter region of key antioxidant genes. In addition, Nrf2 has been shown to play a role in the decrease in inflammation through interaction with nuclear factor kappa B (NFκB) and direct inhibition of IL‐1β and interleukin 18 (IL‐18) cytokine transcription.^51^ Nrf2 is therefore recognised as an important therapeutic target, which is made all the more attractive given its responsiveness to activation by pseudo‐stressors.^52^ These pseudo‐stressors are mostly small molecules derived from natural plants, such as curcumin from turmeric, resveratrol from red wine, and oleanolic acid from olive oil. Several synthetic derivatives have been developed with improved bioavailability and Nrf2‐activating ability.

#### dh404

One potent synthetic derivative of oleanolic acid, capable of strongly activating Nrf2, is 2‐cyano‐3,12‐dioxo‐oleana‐1,9,‐dien‐28‐oic acid (CDDO), also known as bardoxolone methyl (BM) or RTA 401.^53^ Subsequent chemical modifications of CDDO led to the development of the triterpenoid, dihydro‐CDDO‐trifluoroethyl amide (dh404). In a preclinical study using dh404, it could be demonstrated that diabetes‐associated atherosclerosis is significantly attenuated, along with reductions in pro‐inflammatory mediators, MCP‐1, vascular cell adhesion molecule 1 (VCAM‐1) and the p65 subunit of NF‐κB, suggesting that modulations of this pathway by pseudo‐stressors may be a feasible therapeutic strategy to lessen diabetes‐associated CVD.^54^, ^55^

#### Sulphoraphane

Sulphoraphane is an organosulphur compound derived from cruciferous vegetables, such as broccoli, and a natural Nrf2 activator.^56^ It directly interacts with Keap1 by modifying the critical cysteine thiol amino acid residues present on this repressor protein, thereby allowing Nrf2 to translocate into the nucleus to induce the expression of ARE‐containing genes.^56^ A study using rabbits fed a high‐cholesterol diet showed that sulphoraphane reduced high‐density lipoprotein‐cholesterol (HDL‐C), glutathione (GSH) and normalised SOD and NOX in the aorta. Reductions in atherosclerosis were noted along with a decrease in NFκB expression in aortic tissue,^57^ suggesting that Nrf2 activation with sulphoraphane acts to lessen atherogenic processes via inhibition of pro‐oxidative and pro‐inflammatory pathways. A concurrent study demonstrated that direct administration of hydrogen sulphide to STZ treated low‐density lipoprotein receptor (LDLR) knockout (KO) mice, increased S‐sulfhydration of Keap1, resulting in the decreased binding of Keap1 to Nrf2. This enhanced the translocation of Nrf2 into the nucleus, and reduced the levels of diabetes‐accelerated atherosclerosis.^58^

#### Heat‐shock protein 90

Heat‐shock protein 90 (Hsp90) has been shown to be over‐expressed in atherosclerotic plaque and plays a role in sustaining inflammatory mechanisms.^59^ The inhibition of Hsp90 (using 17‐dimethylaminoethylamino‐17‐demethoxygeldanamycin) led to an increase in the activation of Nrf2, and an inhibition of pro‐inflammatory NFκB in atherosclerotic plaques, resulting in reduced lesion size and decreased leucocytes and cytokines.^59^ Furthermore, inhibition of Hsp90 led to increased induction of HO‐1, SOD, catalase and autophagic machinery in the aortic tissue. Considering that Nrf2/Keap1 acts upstream of the pro‐inflammatory IL‐1β signal pathway, and that inhibition of Hsp90 also leads to an increase in Nrf2, the specific targeting of components of the antioxidant/inflammatory pathway are valuable avenues of research for future pharmacological management of atherosclerosis.^49^

In summary, ROS and inflammation are increased in the major cell types of the diabetic vasculature, driven by hyperglycaemia‐induced dysregulation of ROS production and removal. Targeting inflammatory mediators, as well as the Nrf2 pathway, are innovative approaches to lessen the cardiovascular burden associated with both T1D and T2D.

## Diabetic nephropathy, antioxidant defences and immunotherapies

Diabetic nephropathy (DN) is the most common cause of end‐stage renal disease (ESRD) with the disease affecting one‐third of patients with diabetes mellitus.^60^ Early signs of DN are glomerular hyperfiltration, glomerular and tubular hypertrophy, and microalbuminuria.^61^ As DN progresses, there is mesangial matrix expansion and glomerular basement membrane thickening. At the advanced stages of DN, a decrease in glomerular filtration rate (GFR) occurs, along with glomerular sclerosis, proteinuria and the detection of macro‐albuminuria.^62^

### The role of oxidative stress and its modulation in diabetic nephropathy

**Figure 2:**
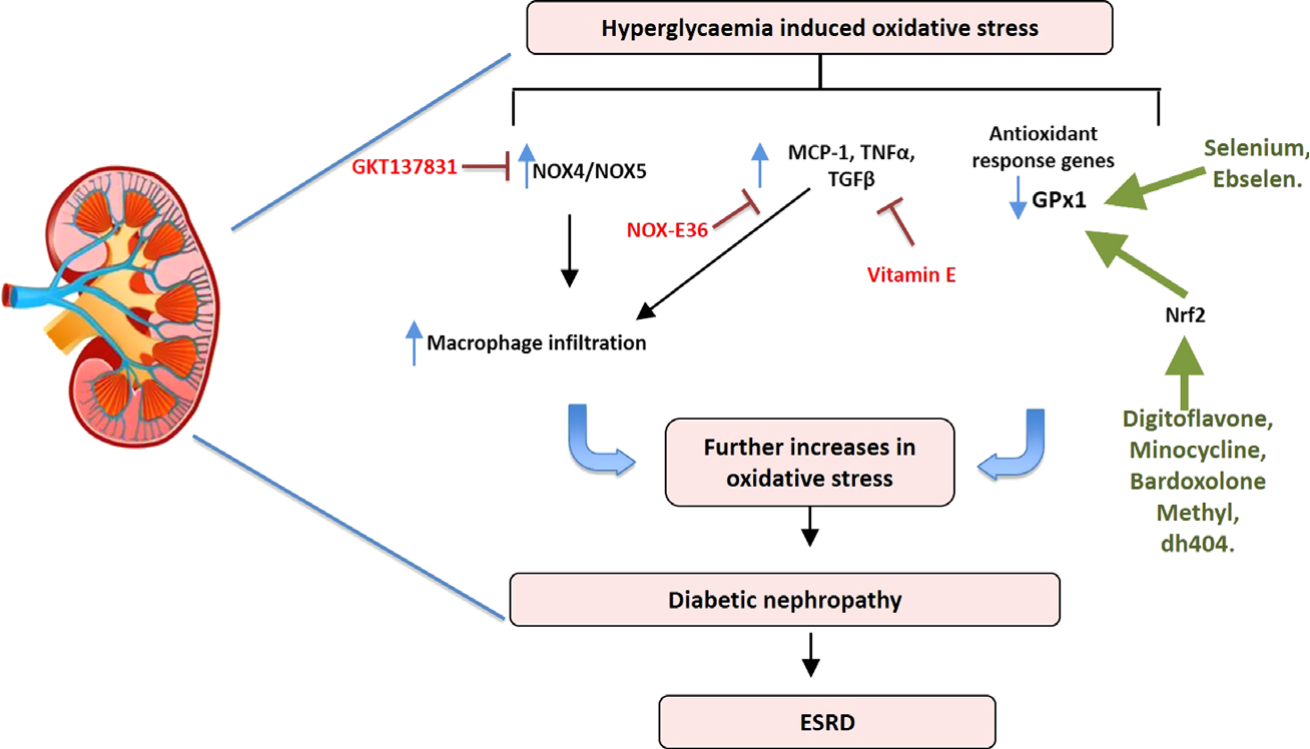
A schematic diagram demonstrating the mediators involved in hyperglycaemia‐induced oxidative stress leading to the progression of diabetic nephropathy. In the kidney, oxidative stress leads to the upregulation of NADPH oxidase (NOX4/NOX5), monocyte chemotactic protein 1 (MCP‐1), tumor necrosis factor alpha (TNF‐α). In red are the inhibitors (GKT137831), Spiegelmer emapticap pegol (NOX‐E36) and vitamin E, whereas in green are antioxidant activators (digitoflavone, minocycline, bardoxolone methyl, dh404, selenium and ebselen) that have shown protective effects in diabetic nephropathy.

Oxidative stress plays a causal role in DN^62^ (Figure 2). The main sources of superoxide in the kidney are the NOX enzymes, specifically the homologs NOX4 and NOX5, the latter only present in humans.^63^ Many factors that are upregulated in diabetes affect the expression and activity of these enzymes including glucose and the RAAS resulting in increases in pro‐inflammatory and pro‐fibrotic markers including the NFκB p65 subunit, transforming growth factor beta (TGF‐β), tumor necrosis factor alpha (TNF‐α) and fibronectin.^64^ The current treatments for DN include glucose control and RAAS blockade; however, these treatments only slow the progression of the disease, rather than improving DN and patient outcome.

Preclinical studies that target oxidative stress to prevent or reverse DN have focused on drugs to either inhibit ROS producing enzymes or increase the expression and/or activity of antioxidants. Recent studies have examined the use of a dual NOX1/NOX4 inhibitor (GKT137831) to prevent the development of DN^65^ in both early and delayed interventional T1D mouse models.^64^ These studies showed that inhibiting NOX1/NOX4 confers renoprotection by decreasing albuminuria, ROS production and glomerular macrophage infiltration.^64^, ^65^ Furthermore, this was associated with the decreased expression of pro‐inflammatory and pro‐fibrotic markers.

Oxidative stress and inflammation concomitantly regulate leucocyte infiltration into the kidney which results in the exacerbation of DN (Figure 2). A key feature of this phenomenon is the increased expression of inflammatory adhesion molecules in the diabetic setting. A recent retrospective autopsy study characterising macrophages in patients with T2D observed that there were macrophage populations present in both diabetic patients without DN and in diabetic patients with proven DN.^66^ Interestingly, there was no difference in the number of glomerular macrophages between the groups. However, after examining the expression of CD68 (a general inflammatory macrophage marker) and CD163 (an M2 or anti‐inflammatory marker) in the glomerulus, there was a positive correlation with CD163+ cells and the severity of DN, whereas an inverse correlation was seen between renal function and CD68+ cells.^66^ Additionally, the observed correlation between the number of interstitial CD68+ macrophages and renal function indicated that macrophages play a role in DN progression.^66^ Since it is known that MCP‐1 can regulate recruitment of macrophages, a recent study by Boels *et al*.^67^ examined the effect of inhibiting MCP‐1 in diabetic mice. MCP‐1 inhibition with Spiegelmer emapticap pegol (NOX‐E36) in diabetic mice resulted in reduced CCR2 expression in circulating monocytes and a reduction in albuminuria, the albumin‐to‐creatinine ratio (ACR) and inflammation without changing the overall number of macrophages in the kidney. Instead, it was observed that diabetic mice receiving NOX‐E36 had a switch in macrophage cytokine profile towards an anti‐inflammatory phenotype.^67^ When NOX‐E36 was given for 3 months in a randomised double‐blind placebo controlled Phase IIa clinical trial, a 29% reduction in ACR was observed with no change to GFR.^68^

Activation and/or enhanced expression of antioxidant defence systems such as GPx‐1, catalase, thioredoxin 1 and 2 (TRX1 and TRX2) or SOD1 and SOD2 is another possible strategy to prevent DN. A recent study using Tempol (a superoxide dismutase mimetic) in a rat model of DN demonstrated attenuated glomerular injury and tubulointerstitial fibrosis in diabetic kidneys.^69^ Additionally, a reduction in MCP‐1 and TNF‐α gene expression and oxidative stress as determined by an attenuation in nitrotyrosine was observed in Tempol treated rat kidneys.^69^ Similarly, the GPx mimetic ebselen, lessened endpoints of DN such as fibrosis and pro‐inflammatory pathways whilst improving kidney function (eGFR) in a mouse model of DN.^70^ The hypoglycaemic drug metformin, a first‐line therapy for T2D patients, reduces ROS and increases the activity of SOD1 and catalase in macrophages, resulting in an increased antioxidant profile.^71^ Furthermore, lipopolysaccharide (LPS)‐induced changes in SOD2, IL‐1β and TNF‐α were attenuated with Metformin treatment.^71^ Although these studies were performed *in vitro*, they give insight into the effect Metformin has on macrophages.

The antioxidant TRX is inhibited by thioredoxin‐interacting protein (TXNIP) both of which have been shown to be upregulated in urine and peripheral blood mononuclear cells (PBMCs) from T1D patients with DN.^72^ In this setting, only TXNIP expression correlated with GFR decline. Interestingly, the angiotensin (Ang)II receptor blocker, telmisartan, demonstrated reno‐protection in a T2D rat model by improving oxidative stress through the downregulation of TXNIP.^73^

Activators of the transcription factor Nrf2 have also been investigated for their effectiveness in DN. One such study investigated the Nrf2 activator digitoflavone in wild‐type (WT) or Nrf2 KO mice. Digitoflavone treatment reduced oxidative stress and inflammation as well as TGF‐β expression in WT mice but not in the Nrf2 KO mice, indicating that Nrf2 was required for the action of digitoflavone.^74^ Interestingly, the potent Nrf2 activator, bardoxolone methyl, was effective against DN in preclinical studies; however, a Phase 3 clinical trial was terminated early due to adverse cardiovascular side effects.^75^ Further investigation into the trial outcome eluded to the fact that fluid overload was observed in a small percentage of at‐risk patients who entered the trial with elevated B‐type natriuretic peptide (BNP) levels, a known biomarker associated with congestive heart failure.^76^ Fluid overload occurred in the first 4 weeks after randomisation and could be minimised with appropriate diuretic treatment. These data suggest the need for more stringent screening of DN patients who are administered Nrf2 activating drugs. Indeed, Phase 2/3 clinical trials for bardoxolone methyl in patients with chronic kidney disease caused by Alport syndrome (the CARDINAL study), with appropriate patient monitoring, are currently progressing. Completion of the Phase 2 study has shown early positive results, with a statistically significant increase in kidney function from baseline at the 12‐week endpoint. A more recent study by Shahzad *et al*.^77^ using the antibiotic minocycline has exhibited antioxidative effects in the kidneys of db/db mice. The action of minocycline was demonstrated to be due to stabilisation of Nrf2 leading to increased protein expression, an effect that was not observed in Nrf2 KO mice. Thus, careful regulation of dose and type of Nrf2 activator is critical for future clinical trials.

### Recent diabetic nephropathy clinical trials

Many recently published clinical trials have examined the effects of selenium, L‐carnosine and Vitamin E to treat/prevent DN. Selenium was shown to prevent oxidative stress markers in DN. Reductions were observed in high sensitivity C‐reactive protein (Hs‐CRP), matrix metalloprotease 2 (MMP2) and increased plasma GPx‐1.^78^ l‐carnosine is a ROS scavenger that was used in a 12‐week double‐blind placebo controlled clinical trial of 90 patients with DN. At the conclusion of the trial, oral carnosine supplementation resulted in reduced oxidative stress, improved glycemic control and renal function.^79^ One of the most well‐known antioxidants is Vitamin E; however, there is limited data on the effects of this vitamin on DN. Recently, a study by Khatami *et al*.^80^ used high dose vitamin E in a randomised double‐blind placebo‐controlled clinical trial of 60 patients. Patients that received vitamin E demonstrated reductions in TNF‐α, MMP2, MMP9 and AGEs, all of which are known to be upregulated in disease settings and contribute significantly to disease progression.^80^ These trials are promising but further investigation is warranted using these compounds in larger patient cohorts.

In summary, there is significant preclinical and clinical evidence for a role for oxidative stress and inflammation in promoting DN. Therapies aimed at these two drivers of DN are showing promise in attenuating structural and functional changes. Targeting the major ROS producers of the diabetic kidney such as NOX4 is showing promise in preclinical studies but awaits further evaluation in clinical trials, whilst the conversion of the macrophage population from an inflammatory into an anti‐inflammatory phenotype is showing some early clinical success. Targeting Nrf2 has improved kidney function in clinical trials, but issues relating to heart failure need careful consideration and patient stratification. With respect to other antioxidants, there is still a paucity of clinical data for DN.

## Diabetic retinopathy, antioxidant defences and immunotherapies

Vascular retinopathies cover a range of diseases spanning both ends of the age‐related spectrum, including neonatal retinopathies, diabetic retinopathies and older degenerative retinal vasculopathies such as macular degeneration. Retinal vasculopathies are a major contributor to impaired vision, leading to blindness. Diabetic retinopathy (DR) creates a major health burden, affecting 93 million people globally.^81^ Approximately 25% of patients with T1D develop retinal vasculopathies after 5 years of diabetes, increasing to 60% after 10 years. Eventually, almost all patients with T1D will develop DR after 25 years of diabetes. Patients with T2D often present with background retinopathy at the time of diagnosis. After 20 years of diabetes, more than 60% of patients will have DR.^81^ Longer diabetes duration and poorer glycemic and blood pressure control are strongly associated with DR.^81^ Treatment options are currently limited and mostly focus on laser therapies to remove damaged tissue.^82^ Additional treatments include anti‐vascular endothelial growth factor (VEGF) to limit new vessel growth.^83^ Understanding the molecular mechanisms involved in driving these vasculopathies is opening up new avenues for a more targeted approach to degenerative retinal therapy.

At the molecular level, the initial primary driver of retinal diabetes is glucose, which affects the same metabolic pathways mentioned previously including the polyol, hexokinase and the PKC pathways, the AGE/RAGE axis and RAAS. In a similar fashion, this affects ROS production and ultimately drives up inflammation. As with all diabetic complications, the mainstay treatment for patients with diabetic retinopathy, is tightly‐regulated blood glucose control, and lipid and blood pressure lowering medication. Yet despite these treatment options, once initiated, retinal damage is progressive and hard to treat. Since oxidative stress and inflammation are linked and are shown to be clear drivers of progressive retinal injury, treatment therapies are now focused on reducing these stressors, with new treatment modalities emerging.

### Targeting oxidative stress in diabetic retinopathy

**Figure 3:**
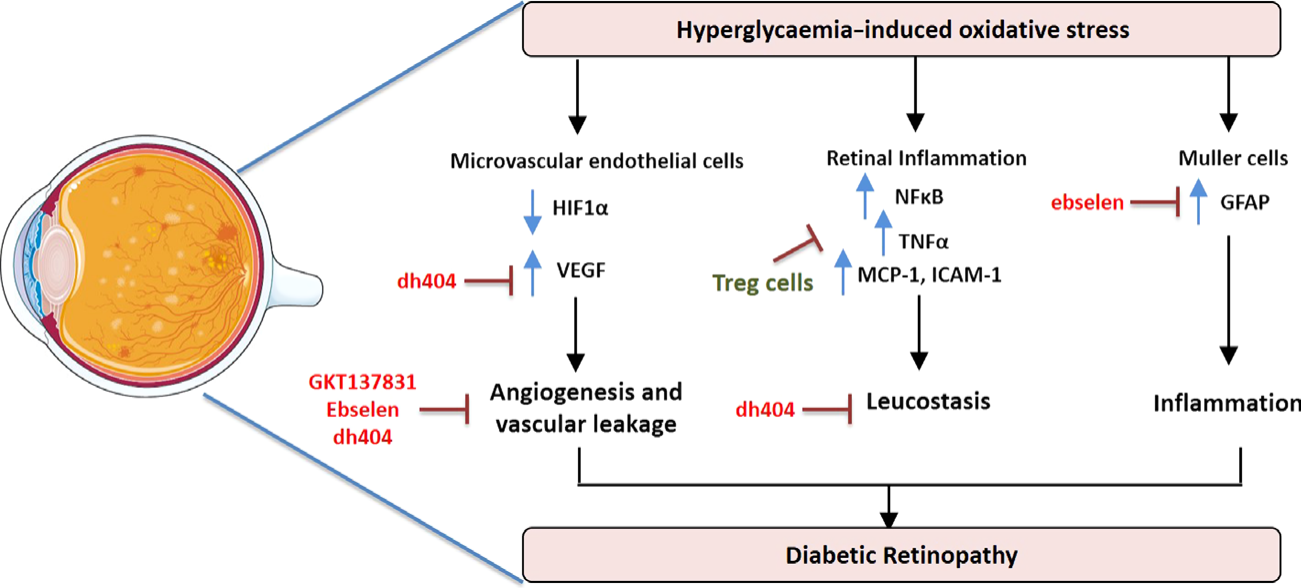
A schematic diagram demonstrating the mediators involved in hyperglycaemia‐induced oxidative stress leading to the progression of diabetic retinopathy. In the microvascular endothelial cells of the retina, oxidative stress leads to decreased expression of hypoxia‐inducible factor alpha (HIF1α) which in turn upregulates vascular endothelial growth factor (VEGF) leading to angiogenesis and vascular leakage. Oxidative stress upregulates retinal inflammation by increasing expression of pro‐inflammatory proteins, for example nuclear factor kappa‐light‐chain‐enhancer of activated B cells (NF‐kB), monocyte chemotactic protein 1 (MCP‐1) and intercellular adhesion molecule 1 (ICAM‐1). In addition, the Müller cells contribute to oxidative stress induced inflammation by upregulating glial fibrillary protein (GFAP). In red are current oxidative enzyme inhibitors (GKT137831) and antioxidant activators (ebselen and dh404) that have shown protective effects in diabetic retinopathy. In green are the T regulatory cells (Treg) recently shown to inhibit retinal inflammation.

The retina is particularly susceptible to oxidative damage^84^ due to a high metabolic rate and the rapid rate of oxygen consumption by the cells of the retina including the rod and cone photoreceptors.^85^ Oxygen consumption rate is generally a readout of mitochondrial function. Recent evidence suggests a limited reserve capacity of the mitochondria of retinal photoreceptors, indicating a state of high metabolic stress, and suggesting that small reductions in mitochondrial ATP production could have negative effects on retinal homeostasis.^86^ In DR, excess ROS damage the microvasculature by affecting the inducible transcription factor, hypoxia‐inducible factor alpha (HIF1α) which in turn upregulates VEGF. This leads to pathological angiogenesis and vascular leakage^87^ (Figure 3). A cyclical amplification of the signal results where VEGF via VEGF receptor 2 and the NOX enzymes leads to further ROS production and additional activation of HIF1α. Furthermore, elevations in ROS activate pro‐inflammatory signals, mostly driven by NF‐κB, which in turn drive up the expression of TNF‐α and downstream increases in inflammatory mediators such as interleukin‐6 (IL‐6), MCP‐1 and ICAM1^88^ (Figure 3).

In preclinical studies, extensive antioxidant focus has been on micronutrients such as vitamins C and E, alpha‐lipoic acid, *N*‐acetyl cysteine (NAC), beta‐carotene and taurine to limit oxidative stress. This has led to reductions in the severity of DR in cell culture experiments and animal models.^89^, ^90^ Clinical trial with antioxidants to treat DR is limited. Future clinical studies using more targeted antioxidant approaches and stratified patient groups are required to fully explore antioxidant therapies in diabetic retinopathy.

Studies of vascular proliferation are not possible in rodent models of diabetes since retinal lesions do not progress to retinal neovascularisation. This limitation has been overcome in recent years with the development of a reliable alternate model of vascular injury. In the oxygen induced retinopathy model (OIR), assessment of neovessel formation is possible in neonatal retinas exposed to two phases of oxygen tension.^91^ In the first phase (Phase I), 7‐day‐old neonatal pups are exposed to high oxygen levels (75%) for 5 days after which removal to room air facilitates a second phase (Phase II) of neoproliferative vascular growth. In Phase I, exposure to high oxygen damages the endothelial cells lining the microvasculature, leading to cell death and vaso‐obliteration of the vascular beds that supply vital blood‐containing oxygen and nutrients to the surrounding tissue. In addition, during this phase, the hyperoxia suppresses VEGF expression, which results in the cessation of normal vessel growth, regression of existing vessels and further contributes to the vaso‐obliteration of the retinal vasculature. During Phase II, hypoxia as a consequence of an inadequate vasculature activates the HIF1α‐VEGF pathway to promote new vessel growth, resulting in neovascularisation with vascular leakage.^91^ Utilising this model has led to significant insight into a role for oxidant stress in driving diabetic neovascularisation. For example, in NOX1, 2 and 4 knockout mice, the NOX1 isoform was shown to be the predominant isoform driving retinal neovascularisation.^92^ Of significance, NOX1 knockout mice showed reduced adherence of inflammatory leucocytes to the vasculature coupled with a lower abundance of the macrophage‐like cells of the retina, namely the microglial cells, suggesting an interplay between reduced oxidative stress and pro‐inflammatory mediators. Aberrant expression of other NOX isoforms may contribute to vascular damage, with NOX4 implicated in vascular endothelial cell damage leading to intravitreal neovascularisation in a rat model of OIR.^93^ Pharmacological inhibitors of the NOX isozymes have shown improvements in capillary degradation, vascular leakage and neovascularisation. In particular, the NOX1/NOX4 inhibitor, GKT137831, in clinical development, is showing promise for DR as well as other diabetic complications such as nephropathy.

Targeting of oxidative stress is also possible by bolstering antioxidant defences. Recent studies have shown a pivotal role for the ubiquitously expressed antioxidant enzyme GPx1 in limiting retinal injury.^94^ Using the OIR model, studies showed that lack of GPx1 led to increased ROS production, significant vaso‐obliteration of the retinal vasculature and intravitreous neovascularisation. At the molecular level, lack of GPx1 caused significant increase in VEGF that was most likely responsible for the modest yet significant, neovascularisation observed in this model. Through the use of a GPx1 mimetic, ebselen, a follow‐up study clearly showed that bolstering antioxidant defences lessened capillary vaso‐obliteration and neovascularisation with a concomitant reduction in cell damage of Müller cells.^95^ Müller cells play a particularly important role in maintaining retinal function as well as vascular integrity. When damaged by oxidative stress, Müller cells become gliotic, meaning that they upregulate the expression of an intermediate filament, glial fibrillary protein (GFAP), and increase inflammatory mediators including cytokines, chemokines and adhesion molecules (Figure 3). In the OIR model as well as in cell culture experiments, ebselen was able to protect Müller cells from gliosis. Given the anatomical association of Müller cell processes with the retinal vasculature, protection of Müller cells by ebselen suggests that this specific antioxidant strategy protects against capillary degeneration and neovascularisation. Furthermore, ebselen was able to upregulate Nrf2‐driven genes, HO‐1, NQO1 and glutamate‐cysteine ligase (GCL) in Müller cells exposed to hypoxia, whilst driving down the expression of pro‐inflammatory genes, IL‐6, MCP‐1 and ICAM‐1.^95^

The oxidative stress regulating transcription factor, Nrf2, is expressed in important cell types of the human retina such as the Müller cells, astrocytes, microglia and ganglion cells, and weakly in the vascular endothelial cells. Importantly, Nrf2 has been shown to play a central role in the protection against OIR in mice.^96^ In particular, via upregulation of downstream targets, Nrf2 protected against the development of diabetic vasculopathies in mice.^97^ In a recent study, using the chemically modified bardoxolone methyl derivative, dh404, it could be shown that upregulation of Nrf2 by dh404 reduces retinal vaso‐obliteration and neovascularisation in phase II of OIR in the mouse.^98^ dh404 also restored the retinal architecture by improving astrocyte‐blood vessel interactions, and limited pro‐angiogenic factors such as VEGF, Müller cell gliosis and vascular leakage. Furthermore, dh404 reduced retinal inflammation, which is significantly increased by OIR, by lessening leucocyte adherence to the vasculature (known as leucostasis). Microglia, the resident immune cells, when activated, release pro‐inflammatory cytokines. dh404 lessened staining for microglia in OIR, suggesting that this strategy is able to limit the presence of pro‐inflammatory effector cells to lessen vascular injury.

### Targeting inflammation in diabetic retinopathy

Recent studies have highlighted the importance of immune cell function in the protection against diabetic retinal injury.^99^ As already discussed, targeting of microglia, which become activated under hyperglycaemic conditions, has been shown to lessen vascular injury. A significant recent finding has been the role that T regulatory cells (Tregs) play in lessening retinal damage associated with OIR. Tregs are important effector cells of the adaptive immune system and maintain immune homeostasis and self‐tolerance through strong immunosuppressive properties. In a study by Deliyanti *et al*.,^100^ it was demonstrated that Tregs migrate to the damaged retina during OIR, and that after manipulations to expand the Treg cell population, prevented vascular damage specifically vaso‐obliteration, neovascularisation and vascular leakage, as well as significantly attenuating VEGF levels. Given the known ability of Tregs to dampen tissue inflammation by specifically lessening macrophage functionality and activity through the release of suppressive cytokines, these results suggest that harnessing the immunosuppressive power of Tregs is a potential unique therapeutic strategy for the treatment of vascular retinopathies.

### Antioxidants and anti‐inflammatory agents to treat diabetic retinopathy

Despite the lack of reliable clinical data in favor of antioxidant therapies, a more targeted approach focussed on particular sources of ROS generation such as the NOX isozymes, or bolstering appropriate antioxidant defences as highlighted above, are some of the more recent and innovative approaches currently being explored preclinically to lessen DR. Importantly, these approaches offer new avenues for therapeutic intervention. In addition, exciting new immunological approaches such as the targeted activation of specific cell subtypes are additional avenues being explored. It is anticipated that these approaches will add to or surpass current treatment modalities to improve outcomes for diabetic patients with these retinal vascular injuries.

## Diabetic cardiomyopathy, antioxidant defences and immunotherapies

In the absence of coronary artery disease and other comorbidities, diabetes increases the risk of developing heart failure by approximately twofold to threefold. This distinct process, termed diabetic cardiomyopathy, was first described over four decades ago. Diabetic cardiomyopathy is characterised by the initial impairment of left ventricular (LV) relaxation, with later impairment of LV contractile function.^101^ The pathophysiological mechanisms underlying diabetic cardiomyopathy are multifactorial and complex, and include an increase in oxidative stress, inflammation, myocardial fibrosis, cardiomyocyte hypertrophy and diastolic dysfunction, as recently reviewed in detail^102^ (Figure 1). However, the sequence in which these aberrations occur, and their relative contribution to overall disease pathology is currently the subject of intense research.

### Role of oxidative stress in diabetic cardiomyopathy

In a similar vein to all diabetic complications, the exposure of cardiomyocytes to elevated glucose causes alterations in metabolism and increased flux through classical oxidative stress pathways, including polyol, hexosamine, AGE/RAGE axis and PKC.^103^ Importantly, changes in flux through these pathways can have detrimental consequences, with the production of mitochondrial ROS, nonenzymatic glycation of proteins and glucose auto‐oxidation. One example is an increased tendency for the formation of reversible Schiff bases and Amadori products following the interaction of glucose with collagen, and the subsequent nonreversible formation of AGEs. Importantly, the presence of cross‐linked collagen in both the vessels and the myocardial tissue has been heavily associated with an increase in diastolic stiffness, indicative of the functional changes witnessed in diabetic cardiomyopathy.^104^

With respect to the activation of PKC, preclinical evidence in the diabetic rat heart has shown that administration of PKC inhibitors is protective against diabetic cardiomyopathy.^105^, ^106^ This is pertinent as insulin resistance leads to the cellular depletion of glucose transporters, GLUT1 and GLUT4, initiating a switch to fatty acid oxidation that ultimately results in reduced cardiac performance. In the cardiomyocyte, acetyl coenzyme A derivatives, the metabolites of free fatty acids, activate PKC and consequently inhibit insulin signalling. The ensuing increase in glucose and insulin further potentiates this vicious cycle of insulin resistance. Similarly, RAAS activation is associated with the presence of insulin resistance and the development of T2D, with angiotensin‐converting enzyme (ACE) inhibitors shown to lessen important hallmarks of diabetic cardiomyopathy, such as oxidative damage, inflammation, cardiomyocyte hypertrophy and apoptosis, and myocardial fibrosis.^107^, ^108^

A study by Clark *et al*.^109^ has highlighted the importance of the hexosamine pathway in diabetic cardiomyopathy by showing that activation of this pathway is significantly increased in cultured cardiomyocytes exposed to high glucose and results in hyperglycaemia‐induced cardiomyocyte dysfunction. Activation of these pathways triggers increased ROS formation, via both mitochondrial and cytosolic sources, leading to altered cardiomyocyte function. Furthermore, NOX is considered one of the major cytosolic sources of ROS production in the diabetic cardiomyocyte.^110^ Numerous studies have shown that there is an increase in NOX4 expression in the cardiomyocytes of OVE26 T1D mice and rats, and in cultured cardiomyocytes exposed to high glucose.^111^, ^112^ Moreover, although no direct *in vivo* evidence is available for NOX2 in mice, this enzyme is suggested to be responsible for high‐glucose‐induced ROS production and cell injury in cultured cardiomyocytes.^110^

### Role of inflammation in diabetic cardiomyopathy

Chronic low‐grade inflammation is associated with diabetes, and evidence has emerged demonstrating that the inflammatory process contributes to the pathogenesis of diabetic cardiomyopathy. Much of the inflammatory response in diabetic cardiomyocytes is attributed to activation of the transcription factor NF‐κB by elevations in ROS,^113^ although increased levels of cytokines, chemokines, adhesion molecules and infiltrating leucocytes are also important in this process. In experimental STZ‐induced T1D models of diabetic cardiomyopathy, NF‐κB activation was shown to be correlated with the increased release of IL‐1β and TNF‐α.^113^, ^114^, ^115^ Moreover, elevated levels of these two pro‐inflammatory cytokines have been found in the circulation and hearts of patients with T2D.^116^ In fact, several studies report a strong correlation between the characteristic hallmarks of diabetic cardiomyopathy and the levels NF‐κB and cytokines.^113^ It should be noted that cardiomyocytes and fibroblasts express receptors for IL‐1β and TNF‐α. Upon binding of IL‐1β and TNF‐α to their respective receptors, a signal transduction pathway is activated which stimulates NF‐κB, resulting in a positive feedback loop that contributes to the intensification of diabetes‐induced inflammation in the myocardium. Once activated, NF‐κB, additionally drives the expression of numerous growth factors, such as connective tissue growth factor (CTGF) and TGF‐β.^113^ This, in turn, drives the production of extracellular matrix (ECM) proteins in the heart, including collagen, fibronectin and proteoglycans, leading to fibrosis, LV remodelling and stiffness, and reduced relaxation of the heart.^108^, ^117^ Moreover, a clinical study showed a correlation between macrophage inhibitory factor and the development of diastolic dysfunction in diabetic patients.^116^ This is of interest as one of the glucagon‐like peptide 1 (GLP‐1) agonists, exendin‐4, a drug class primarily employed in the clinic due to its inherent ability to lower blood glucose, was shown to attenuate adverse cardiac remodelling in diabetic mice, specifically by reducing inflammation, independent of its known glucose‐lowering actions.^118^

### Current clinical interventions for the treatment of heart failure

Several clinical interventions are currently available and used for treating heart failure in both nondiabetic and diabetic patients. Three common therapies include the use of angiotensin‐converting enzyme inhibitors, beta‐blockers (β‐blockers) and calcium channel blockers (CCBs). However, despite their common use in heart failure management, none of the therapies have been specifically designed to treat heart failure caused as a direct result of diabetes. Previous clinical strategies have focused on more general antioxidant therapies based on the clear preclinical evidence of a role for oxidative stress and inflammation in the development of diabetic heart failure. However, these unfocused vitamin strategies failed to translate into useful clinical therapies to reduce cardiovascular morbidity and mortality.^119^, ^120^ The focus is now on therapeutics with the ability to target‐specific focal ROS, strategies to improve endogenous antioxidant responses, and novel compounds to lessen inflammation.

#### Nrf2 activators

Attention has focused on the transcription factor Nrf2 after it was established that ROS levels were significantly increased in neonatal and adult cardiomyocytes obtained from diabetic Nrf2 knockout mice, suggesting that Nrf2 is a critical regulator of defence against ROS in diabetic hearts.^121^, ^122^ In addition, studies have also shown that Nrf2 responds to pro‐inflammatory stimuli and protects cells from inflammatory damage, albeit in cell types such as vascular endothelial cells.^123^ Evidence in support of known activators of Nrf2 in limiting diabetic cardiac injury is discussed below.

#### Sulphoraphane

A study using STZ‐induced T1D mice showed that sulphoraphane attenuates cardiac dysfunction and remodelling after 3 months of treatment.^124^ Furthermore, this cardioprotective effect persisted for 3 months after treatment ended.^124^ In a study of T2D mice, sulphoraphane treatment significantly attenuated cardiac remodelling and dysfunction by reducing cardiac lipid accumulation, cardiac inflammation and oxidative stress and fibrosis.^125^ In this study, sulphoraphane, via upregulation of Nrf2‐mediated downstream genes, NQO1 and HO‐1, reversed the diabetes‐induced inhibition of the liver kinase B1 (LKB1)/activated protein kinase (AMPK) signalling pathway, thereby preventing diabetes‐induced lipotoxicity and cardiomyopathy.^125^ A subsequent study has shown the downstream involvement of other antioxidants including metallothionein in the Nrf2‐mediated cardiac protection.^126^

#### Resveratrol

Resveratrol is a polyphenolic natural Nrf2 activator found in the skins of grapes and berries. Resveratrol downregulates Keap1 expression.^127^ In the STZ‐induced T1D rat kidney, resveratrol significantly restored the activity of several antioxidants and decreased oxidative stress.^128^ Resveratrol was shown to activate Silent information regulator 1 (Sirt 1), a deacetylase protein, and alleviate cardiac dysfunction via improved mitochondrial function in diabetic mice and in cardiomyocytes treated with high glucose.^129^ Importantly, the resveratrol‐mediated improvements were shown to be mediated via peroxisome proliferator‐activated receptor gamma coactivator 1‐alpha (PGC‐1α)‐mediated mitochondrial regulation.^129^ Others have shown that SIRT‐1 activation by resveratrol leads to deacetylation of both NFκB‐p65 and histone 3.^130^ Additionally, resveratrol has recently been reported to ameliorate cardiac dysfunction by inhibiting apoptosis via the phosphoinositide 3‐kinase (PI3K)/protein kinase B (Akt)/forkhead box O3a (FoxO3a) pathway^131^ and to lessen myocardial fibrosis by inhibiting the ROS/extracellular signal‐regulated kinase (ERK)/TGF‐β/periostin pathway in STZ‐induced diabetic mice.^131^ The therapeutic potential of resveratrol on diabetes‐associated complications in humans has not been reported so far.

#### Mg123

MG132 is a less well‐known Nrf2 activator. Its major mechanism of action is through interference of the ubiquitin‐proteasome system (UPS) as a proteasome inhibitor. It exhibits cytoprotective effects by inhibiting the proteasomal degradation of Nrf2, enabling Nrf2 nuclear accumulation and the transduction of Nrf2 target genes. In a study by the Cai laboratory, diabetic OVE26 mice treated with MG132 displayed attenuated diabetes‐associated cardiac complications by upregulating Nrf2 activity and downstream antioxidant genes.^132^ In addition, MG123 functions to lessen inflammation by dampening the activation of the pro‐inflammatory transcription factor NF‐κB. NF‐κB is inactivated through binding with its negative regulator, IκB. IκB is, however, degraded by proteasome ubiquitination. In the same study, Wang *et al*.,^132^ demonstrated that MG123 blocked the degradation of IκB, making IκB available as a repressor of NF‐κB, to provide anti‐inflammation function, ultimately leading to cardiac protection from ischaemic damage. Hence, this strategy of proteasome inhibition is of significance as a therapeutic since specific inhibition of NF‐κB is known to be cardioprotective.

#### Bardoxolone methyl

This recently investigated antioxidant‐inflammatory modulator has shown improvements in diabetes‐associated cardiovascular diseases,^54^ but little is known preclinically about its protective role in diabetic cardiomyopathy. In a model of postmyocardial infarct (MI) remodelling in rats, recent data support a role for dh404 in the protection against adverse ventricular remodelling, as assessed 1 month post‐MI.^55^ In support of a protective role for Nrf2 activation in diabetic cardiomyopathy, a recent study showed that administration of an miR‐144 antagomir (microRNA‐144 drives up oxidative stress and apoptosis of diabetic cardiomyocytes via an Nrf2‐associated pathway), lessened oxidative stress, cardiomyocyte apoptosis and improved cardiac function in STZ‐induced diabetic mice, suggesting a role for the targeting of NRF2 via miR‐144 antagonism as a novel strategy to lessen diabetic cardiomyopathy.^133^

### Anti‐inflammatory drugs

Targeting inflammation in the diabetic heart is a relative new area with preclinical studies now showing promise using novel targeting strategies. This is based on recent evidence that cardiac inflammation is a key contributor to myocardial damage in the diabetic heart.^134^ Interventions that target cardiac inflammation may ultimately limit progression to heart failure and death in diabetes‐affected patients. One strategy involves the targeting of Annexin A1 (ANXA1), an endogenous glucocorticoid‐regulated anti‐inflammatory molecule that limits and resolves inflammation.^135^ ANX‐A1 binds to and activates the family of formyl peptide receptors (G protein‐coupled receptor family) to inhibit neutrophil activation, migration and infiltration. The physiological importance of this molecule was demonstrated in diabetic ANXA1 knockout mice who exhibit a worse diabetic phenotype and develop more severe cardiac dysfunction.^135^ Interestingly, plasma levels of ANXA1 were recently shown to be elevated in individuals with type 1 diabetes, with and without nephropathy compared with healthy individuals, and hypothesised to be elevated as a compensatory mechanism to protect tissues from the deleterious effects of hyperglycaemia.^135^ More recent studies have shown that targeting ANXA1 to increase its expression can protect the diabetic heart from inflammatory injury. Indeed, administration of human recombinant ANXA1 halted further decline in cardiac function in diabetic mice via a pathway that included the restoration of Akt signalling.^135^ Targeting strategies have additionally included the use of ANXA1 peptides^136^ and small‐molecule‐biased formyl peptide receptor agonists to protect against myocardial injury.^137^ Recent data have shown, albeit not in a diabetic context, that myocardial dysfunction postischaemia is prevented by endogenous annexin‐A1 and an N‐terminal‐derived peptide Ac‐ANX‐A1(2‐26).^138^ The anti‐inflammatory properties of annexin‐1 peptides have been largely ascribed to their powerful antineutrophil actions *in vivo*. Given that annexin‐A1 facilitates the resolution of inflammation, it represents an exciting target for the cardiac complications of diabetes.

Another strategy has recently indirectly shown the benefit of targeting inflammation in diabetic cardiomyopathy. Use of dapagliflozin, an SGLT2‐inhibitor and glucose‐lowering drug, was shown to attenuate the deterioration of heart function and lessen activation of the major cytosolic receptor involved in inflammatory cytokine processing, the NLRP3 inflammasome, in diabetic mice. These effects could additionally be augmented with saxagliptin, a DDP4‐inhibitor. Interestingly, these effects were independent of any glucose‐lowering effects of the drugs, suggesting that these drugs may have direct inhibitory effects on pro‐inflammatory pathways.^139^

In summary, several promising novel targeted antioxidant and anti‐inflammatory therapies for diabetic cardiomyopathy are now under preclinical development^102^ including the targeting of the Nrf2 transcription factor. Several of the newer approaches such as annexin A1 targeting are yet to be translated into clinical trials, whilst the positive additional benefits of glucose‐lowering drugs such as dapagliflozin will make this strategy attractive for diabetic patients.

## Conclusions and future perspectives

It is becoming clear that oxidative stress and inflammation are significant risk factors for the development and sustained cellular injury of T1 and T2 diabetic complications. This holds true for all diabetic complications, including diabetes‐associated atherosclerosis with its underlying endothelial dysfunction, diabetic nephropathy, diabetic retinopathy and diabetic cardiomyopathy.

The mechanisms and pathways involved in the response to oxidative stress are more complex than previously appreciated. One of the challenges facing the field of oxidative stress therapeutics is the relative paucity of therapeutic targets that have translated from the laboratory to the clinic. Antioxidants have not yielded cardiovascular benefits in large clinical trials, thus lessening enthusiasm for conventional drug trials aimed at lowering oxidative stress. However, newer strategies which target particular abnormalities in the diabetic milieu are likely to lead to positive results. For example, this review has highlighted that targeting the source of ROS production, (particularly the NOXs), bolstering antioxidant defences (especially the master regulator of oxidative stress, Nrf2) and limiting inflammation (e.g. targeting cytokines as in the CANTOS trial or increasing inflammatory resolving molecules such as annexin‐A1) offers newer avenues for therapeutic intervention. The positive findings of the CANTOS trial, particularly within the diabetic subgroup, provide hope that targeting inflammation is relevant not only to classical anti‐inflammatory disorders but additionally to chronic conditions associated with low‐grade inflammation such as atherosclerosis and/or Type 2 diabetes. Additionally, newer drugs such as the SGLT inhibitors and GLP‐1 agonists are showing added anti‐inflammatory benefits and potentially also may influence oxidative stress. This suggests that the newer glucose‐lowering drugs are likely to confer added end‐organ protection, independent of glucose‐lowering in diabetic patients. It is anticipated that these approaches will result in translatable therapies, that together with currently available drugs, will lessen the burden of these diabetic complications.
